# Gene Therapy for Mucopolysaccharidosis Type II—A Review of the Current Possibilities

**DOI:** 10.3390/ijms22115490

**Published:** 2021-05-23

**Authors:** Paweł Zapolnik, Antoni Pyrkosz

**Affiliations:** 1 Students’ Scientific Association of Clinical Genetics, Department of Clinical Genetics, Medical College, University of Rzeszów, 35-959 Rzeszów, Poland; 2 Department of Clinical Genetics, Medical College, University of Rzeszów, 35-959 Rzeszów, Poland; antoni.pyrkosz@gmail.com

**Keywords:** mucopolysaccharidosis II, Hunter syndrome, adeno-associated viruses, genetic therapy, gene editing, review

## Abstract

Mucopolysaccharidosis type II (MPS II) is a lysosomal storage disorder based on a mutation in the *IDS* gene that encodes iduronate 2-sulphatase. As a result, there is an accumulation of glycosaminoglycans—heparan sulphate and dermatan sulphate—in almost all body tissues, which leads to their dysfunction. Currently, the primary treatment is enzyme replacement therapy, which improves the course of the disease by reducing somatic symptoms, including hepatomegaly and splenomegaly. The enzyme, however, does not cross the blood–brain barrier, and no improvement in the function of the central nervous system has been observed in patients with the severe form of the disease. An alternative method of treatment that solves typical problems of enzyme replacement therapy is gene therapy, i.e., delivery of the correct gene to target cells through an appropriate vector. Much progress has been made in applying gene therapy for MPS II, from cellular models to human clinical trials. In this article, we briefly present the history and basics of gene therapy and discuss the current state of knowledge about the methods of this therapy in mucopolysaccharidosis type II.

## 1. Introduction

### 1.1. The Basics

Mucopolysaccharidosis type II (MPS II, OMIM #309900), also called Hunter syndrome, is a rare monogenic disease belonging to the group of lysosomal storage disorders (LSDs). The estimated incidence of MPS II is 0.3–0.7/100,000 births [1]. The condition occurs more often in the countries of East Asia than in Europe [2]. It is inherited in an X-linked recessive way as the only form among mucopolysaccharidoses. Almost exclusively males are affected, but there have also been reports of affected females, mainly due to non-random inactivation of the X chromosome [3,4,5,6,7,8,9]. Charles A. Hunter first described this entity in 1917 in two brothers [10].

MPS II is caused by a mutation in the *IDS* gene, located at Xq28 (OMIM *300823), which encodes iduronate 2-sulphatase. This enzyme is responsible for catalysing the hydrolysis of sulphate groups from dermatan sulphate (DS) and heparan sulphate (HS) molecules. Enzyme deficiency or decreased activity results in the accumulation of glycosaminoglycans (GAGs) in various tissues and organs, leading to dysfunction [11,12]. Dermatan sulphate and heparan sulphate accumulated in cells disrupt cellular processes such as endocytosis, ion balance, and cell movement. In addition, heparan sulphate promotes the accumulation of GM2 and GM3 gangliosides in the brain, due to which microglial cells are stimulated, and an inflammatory reaction occurs in the central nervous system (CNS) [13]. On the other hand, studies on model organisms (*Danio rerio*, *Mus musculus*) indicate that a mutation in the *IDS* gene may also affect the developmental process differently by influencing the signalling pathway of the fibroblast growth factor (FGF) [14]. Another factor contributing to the development of inflammation is the presence of incompletely degraded GAGs, which may structurally resemble lipopolysaccharide, an endotoxin of Gram-negative bacteria that activates the Toll-like receptor 4 (TLR4). This process leads to the secretion of pro-inflammatory cytokines and the activation of the STAT1/STAT3 protein pathway, increasing the concentration of tumour necrosis factor α (TNF-α) and inflammation in the affected tissues [15].

The animal models, mainly mouse models, of mucopolysaccharidosis type II significantly contributed to understanding the pathophysiology and the application of therapy in this disease, including gene therapy [13]. The first preclinical attempts of gene therapy were carried out in the 1990s [16]. It was also when the first clinical trials began, which resulted in the further development of Hunter syndrome treatment.

### 1.2. Clinical Features

Despite its heterogeneity, the disease is usually classified into two main forms: attenuated (without central nervous system involvement) and severe (with central nervous system involvement). Both conditions show signs of many organs’ dysfunction, such as hepatosplenomegaly, coarse facial features, skeletal system abnormalities (dysostosis multiplex), joint stiffness, short stature, carpal tunnel syndrome, heart valve disease, hypertension and other cardiac abnormalities, communicating hydrocephalus and hearing loss. In addition, patients with MPS II also suffer from frequent respiratory infections and decreased exercise tolerance. The severe form of the disease, which accounts for about 60% of cases, is characterized by the child’s normal development until about 2–4 years of age when cognitive functions deteriorate and the process of acquiring new skills is inhibited. Attention difficulties are also common in school-age patients with the severe form of Hunter syndrome. An additional manifestation of CNS damage is frequent epileptic seizures. Patients with deletions, recombination, frameshift mutations, or splicing abnormalities are more likely to develop a severe phenotype. In the case of missense mutations, some may lead to a severe phenotype but most likely predisposed to the attenuated form. Due to the heterogeneity, in many cases, it is difficult to determine the exact genotype/phenotype correlation and predict the nature of development in a particular patient [13,17].

### 1.3. Diagnosis and Management

For the final diagnosis of the disease, biochemical and molecular tests are performed. First, the level of glycosaminoglycans in a 24-h urine collection is determined and then the activity of the iduronate 2-sulphatase enzyme, e.g., in peripheral blood lymphocytes, skin fibroblasts or chorionic cells (as prenatal diagnosis) is assessed [11,13]. Sanger sequencing is usually performed to detect mutations in the *IDS*, but the development of Next Generation Sequencing (NGS) has paved the way to use gene panels to detect the mutation quickly and exclude other lysosomal storage disorders [11,18,19].

Patients with mucopolysaccharidosis type II, as a disease with a heterogeneous clinical picture, require multidisciplinary care. Historically, before the pathophysiology of the disease was known, management was limited to symptomatic treatment and palliative therapy. In studies conducted on fibroblasts collected from patients with mucopolysaccharidosis type I (MPS I) and mucopolysaccharidosis type II, normal levels of GAGs were observed. Researchers discovered the phenomenon, so-called cross-correction, i.e., enzyme secretion by some cells and its uptake by others via the mannose-6-phosphate receptor [20,21]. The discovery of this phenomenon contributed to the acceleration of studies on therapeutic agents. Currently, the mainstay of treatment is enzyme replacement therapy (ERT) using human recombinant iduronate 2-sulphatase administered intravenously [1]. Thanks to ERT, it is possible to reduce the concentration of GAGs in the urine, reduce the size of the liver and spleen, and improve physical tolerance and stabilize the bone and cardiac abnormalities.

Unfortunately, the enzyme itself cannot cross the blood–brain barrier (BBB). Attempts are made to administer the enzyme intrathecally or intraventricularly. These studies were successfully performed in the MPS II mouse model. Higuchi et al. [22] showed a reduced concentration of GAGs in the brain tissue of mice and other organs, such as the heart, lungs, kidneys, testes, and liver. In addition, they demonstrated an improvement in short-term memory and learning skills, and in brain autopsy, a reduction in cell vacuolization and the expression of the lysosomal marker protein LAMP2. Similar research results were obtained by Sohn et al. [23]. They additionally showed a correlation between the concentration of HS in the cerebrospinal fluid (CSF) and the GAGs levels in the brain. At a later stage, clinical trials were carried out in MPS II patients with the administration of the enzyme via the intrathecal or intraventricular route, along with the standard intravenous drug administration [24]. In a study conducted by Muenzer’s group [25], the enzyme administered intrathecally in increasing doses made it possible to reduce the concentration of GAGs in CSF by 80–90%. Another clinical trial was conducted by the group of Seo et al. [26] in Japan (JMACCT CTR JMA-IIA00350) on a group of six patients with severe MPS II. They were administered idursulfase beta into the brain’s lateral ventricle via a CSF reservoir system implanted under the scalp. The drug was administered in escalating doses from 1 to 30 mg every four weeks from 0 to 24 weeks and then 30 mg to 100 weeks of the study. At the same time, during the study, patients continued to receive the enzyme intravenously. The authors assessed the developmental age based on the Kyoto Scale of Psychological Development 2001 (KSPD). Patients receiving intraventricular idursulfase beta had stabilized CNS function decline compared to patients receiving the enzyme intravenously only. In addition, HS level in CSF at week 100 was significantly reduced compared to the baseline. Anti-idursulfase antibodies were not detected in cerebrospinal fluid during the trial. Apart from a few side effects (pyrexia, vomiting, and upper respiratory tract infection), the therapy was generally well tolerated. Thanks to this study, intraventricularly administered idursulfase beta was approved in Japan as Hunterase^®^ [26]. The results of these studies are promising, but the procedure is more invasive than intravenous injection and still requires repeated administration of the enzyme.

Another way to bypass the difficulties associated with the blood–brain barrier is to create a system that allows the drug to pass through the barrier. For this purpose, two solutions are tried: receptor-mediated transcytosis and the use of carriers [24,27]. The first option uses a natural process by which proteins enter the central nervous system. The enzyme is conjugated with an antibody against a specific receptor and can defeat the BBB by transcytosis via epithelial cells. Clinical trials have been conducted using iduronate 2-sulphatase combined with an anti-human transferrin receptor antibody (J-Brain Cargo^®^, JR-141, JCR Pharmaceuticals) [28] and an anti-insulin receptor antibody (AGT-182, ArmaGen Technologies). The first outcomes of studies with JR-141 showed a positive result in reducing the concentration of HS and DS in the cerebrospinal fluid, urine, and plasma. The positive results of clinical trials conducted in Japan with pabinafusp alfa (JR-141) [29] enabled the approval of Izcargo^®^, a system based on J-Brain Cargo^®^ containing 10 mL of pabinafusp alfa administered intravenously. It is the first agent on the market that allowed the enzyme to cross the blood–brain barrier [30]. The results of using AGT-182 have not been published yet [24]. A clinical trial is currently underway using an enzyme conjugated to an antibody binding site to the transferrin receptor (DNL-310, Denali Therapeutics). There are 16 patients with Hunter syndrome between the ages of 2 and 18 in the study, and recruitment is still open (ClinicalTrials.gov Identifier: NCT04251026) [31]. Another possibility to overcome the BBB is using a carrier that will deliver the enzyme to the cell through the surface heparan sulphate receptor. Such a system using a recombinant enzyme and a guanidinylated neomycin molecule, which has a high affinity for heparan sulphate proteoglycans, has so far been used in a mouse model of mucopolysaccharidosis type I (MPS I). Still, no trials have been carried out on MPS II models [32]. An additional limitation of ERT is the enzyme immunogenicity, against which antibodies in the IgG class are produced, even in about 50% of treated patients [11,33].

Another method of causal treatment is hematopoietic stem cell transplantation (HSCT). Peripheral blood monocytes can cross the blood–brain barrier and settle in the central nervous system as microglial cells [34]. This property, combined with a single transplant procedure, is a strong argument for the use of HSCT. However, complications associated with immunosuppressive therapy and graft versus host disease (GvHD) necessitate careful consideration of this method.

An alternative to the above therapeutic methods is the constantly developing gene therapy. Mucopolysaccharidosis type II is a monogenic disease with relatively well-known pathomechanisms, making it a good candidate as a target for gene therapy. In addition, it allows avoiding the characteristic difficulties of ERT and HSCT. A comparison of the three main treatments in MPS II is shown in Table 1. A more detailed discussion of the current possibilities of gene therapy in MPS II is provided in the next part of the review.

## 2. Gene Therapy

### 2.1. The Basics

The term “gene therapy” includes the use of nucleic acids to treat human disease. Gene therapy is generally divided into in vivo therapy, with the direct administration of the vector to deliver the correct gene to the cells, and ex vivo therapy, in which cells are collected from the patient’s body, applied with the vector and re-administered to the patient. Vectors are classified as viral (e.g., retroviruses, lentiviruses, adeno-associated viruses) and non-viral (e.g., Sleeping Beauty transposon system, nanoemulsions) [35,36]. Gene therapy was first used in the early 1990s in severe combined immunodeficiency due to adenosine deaminase deficiency [37]. The first available agent on the market was Gendicine^®^, an adenovirus with a human gene encoding p53 protein, introduced in 2004 in China, used in squamous cell carcinoma of the head and neck [38,39]. The first gene therapeutics approved in Europe was alipogene tiparvovec (Glybera^®^), introduced in 2012. It is an adeno-associated virus (AAV) vector expressing the human lipoprotein lipase gene used in familial lipoprotein lipase deficiency [40,41]. Currently, numerous clinical trials are conducted in many diseases, and several other drugs are available on the market, including voretigene neparvovec (Luxturna^®^) in congenital retinal dystrophy, onasemnogen abeparvovec (Zolgensma^®^) in spinal muscular atrophy or Strimvelis^®^ in severe combined immunodeficiency caused by adenosine deaminase deficiency [42].

### 2.2. First Attempts Gene Therapy with Retroviruses

The first attempts to create gene therapy for Hunter syndrome were made in the 1990s using retroviral vectors. Initially, good results were obtained in preclinical trials using lymphocytes and hematopoietic stem cells CD34+ [16,43]. Braun et al. [16] used a Moloney murine leukaemia virus-derived retroviral vector containing an *IDS* complementary DNA (cDNA) under the transcriptional control of a long terminal repeat sequence or cytomegalovirus early promoter. The vector was applied to peripheral blood lymphocytes collected from affected persons. The authors assessed the normal activity of iduronate 2-sulphatase and observed the phenomenon of cross-correction between cells. A similar study using a modified virus was carried out by Hong et al. [43] using human hematopoietic cells as target cells. The *IDS* was highly expressed in cells. As a result, a clinical trial was opened but was discontinued due to insufficient gene expression and side effects [44,45]. The limitation of retroviruses is their integration into the host genome, the risk of mutagenesis and immunogenicity. However, research using other viral vectors on model organisms was continued and showed promising results [46,47,48]. Further clinical trials are conducted using various forms of gene therapy, including genome editing systems such as zinc finger nucleases (ZFNs) or Clustered Regularly Interspaced Short Palindromic Repeat/Cas9 (CRISPR/Cas9) [48].

### 2.3. Gene Therapy with Lentiviruses

One of the types of gene therapy is the ex vivo method of collecting cells from the patient to be treated. Usually, for this purpose, laboratories use hematopoietic stem cells or so-called induced pluripotent stem cells (iPSCs) obtained from already differentiated somatic cells, e.g., fibroblasts. iPSCs can later differentiate into many cell types, e.g., neurons, myocytes, hepatocytes, etc. [48,49]. After the cells are harvested, the appropriate genetic modification is made to produce and secrete the correct enzyme. For this purpose, lentiviruses (derived from retroviruses) are usually used, which provide stable gene expression and deliver the missing gene to non-dividing cells, e.g., neurons [50,51]. After the vector has been inserted, the cells are administered to the patient (autologous transplantation) [48]. This form of therapy was successfully applied in the MPS II mouse model by Wakabayashi et al. [52]. The administration of hematopoietic stem cells with the lentiviral vector containing the *IDS* to mice led to an increase in the activity of iduronate 2-sulphatase and a decrease in the accumulation of glycosaminoglycans in the central nervous system and other organs. In addition, the authors demonstrated a reduction in the accumulation of secondary substances related to autophagy, the p62 protein, and protein and ubiquitin conglomerates in the cells. Neither was there any deterioration in neuronal function [52]. As discussed above, lentiviral vectors have the advantages that make them frequently used in gene therapy. However, there is one major limitation. They integrate into the cell’s genome in a random, non-targeted manner, which is associated with the potential risk of mutagenesis and neoplastic transformation. There are currently two clinical trials with this method in mucopolysaccharidosis type I and mucopolysaccharidosis type IIIA (MPS IIIA), but no clinical trials have been undertaken in Hunter syndrome [53,54].

### 2.4. Gene Therapy with Adeno-Associated Viruses

Another way to deliver the correct gene to cells is in vivo gene therapy with adeno-associated viruses (AAVs). They are small viruses (25 nm) belonging to the *Parvoviridae* family. They contain a 4.7 kbp genome as a single-stranded DNA molecule [55]. These viruses cannot replicate on their own and need assistance from another virus, such as an adenovirus or a herpes virus [56]. Human infection with AAVs occurs naturally. The first contact with the wild type of this virus appears around 1–3 years of age. Thus far, it has not been shown that they cause pathology in humans [57,58]. Unlike retroviruses and lentiviruses, they do not integrate with nuclear DNA but function in the form of extra-chromosomal material—episomes. This property reduces the potential risk of mutagenesis. They provide stable expression of the transgene in both dividing and non-dividing cells [11,48,59,60,61]. The limitation of the AAVs is immunogenicity and the risk of generating neutralizing antibodies. Still, of all currently available viral vectors, these appear to be the most advantageous and are most widely used in preclinical studies and clinical trials.

One of the first studies on using of AAVs in lysosomal storage disorders, including mucopolysaccharidoses, was carried out at the end of the 20th century by research groups led by Sands [62] and Davidson [63,64]. They conducted studies on mouse models, obtaining improvements in somatic symptoms and central nervous system function [65].

Cardone et al. conducted the first study using AAVs in an animal model (mouse) of mucopolysaccharidosis type II [66]. The authors used AAV type 2/8 with an inserted cDNA for the human *IDS* gene, under the transcriptional control of the liver-specific promoter of the *TBG* gene. Adult mice were injected intravenously with 1.0 × 10^11^ virus particles. The mice were evaluated for seven months. The authors showed that iduronate 2-sulphatase activity was restored in plasma and tissues (including the central nervous system), and the levels of GAGs in urine were normalized. Skeletal abnormalities also improved. A similar study on a mouse model was carried out by Jung et al. [47], who used the same vector—AAV type 2/8. In this study, the authors introduced *IDS* cDNA into the pAAV2-EF-eGFP-WPRE-polyA plasmid as a substitution for the eGFP element. As a result, they created pAAV2-EF-hIDS-WPRE-polyA plasmid, which, in addition to the coding sequence for iduronate 2-sulphatase, contains the human elongation factor 1-a promoter (EF), woodchuck hepatitis virus posttranscriptional element (WPRE) and a signal sequence of bovine growth hormone poly(A) chain. As in the study mentioned above, the viruses were administered intravenously at a dose of 1.0 × 10^11^ virus particles. The activity of iduronate 2-sulphatase and the concentration of GAGs were assessed 6 and 24 weeks after applying AAVs. Significantly increased activity of the enzyme and decreased concentration of GAGs in the liver, brain, kidneys and spleen have been demonstrated. Additionally, the authors observed a reduction in cell vacuolization in the histopathological examination of the brain [47].

Another type of adeno-associated virus, AAV type 9, was used in subsequent preclinical studies [67,68]. Motas et al. [69] focused on assessing the improvement in the central nervous system functions. They used AAV9 with the coding sequence of the *IDS*, under the control of the ubiquitous CAG promoter. In this case, a mouse model was also used, but the viruses were administered directly into the cerebrospinal fluid by injection into the cisterna magna at a dose of 5.0 × 10^10^ vector particles. Four months after the virus administration, the authors assessed the effects of the therapy. Iduronate 2-sulphatase activity in the central nervous system was determined to be 40% of that in wild-type mice. In addition, they showed that most vectors are located in neurons and not in microglia or astrocytes. The concentration of GAGs and the gene expression profile in the brain was normalized. In addition, the vectors permeated the blood and led to the improvement of somatic symptoms and the accumulation of GAGs in peripheral organs such as the liver, lungs and heart. The authors observed an improvement in motor and cognitive functions and increased overall survival compared to untreated individuals [69].

Promising results from preclinical studies have opened the way to first clinical trials using AAVs to improve central nervous system function in patients with Hunter syndrome. Two multicentre (United States, Brazil) phase I/II clinical trials are currently underway on RGX-121 (Regenxbio Inc.), containing a recombinant AAV9 vector with a cassette expressing the human iduronate 2-sulphatase gene (AAV9.CB7.hIDS) [70,71,72,73].

The first study (ClinicalTrials.gov Identifier: NCT03566043) included patients with the severe form of MPS II, with central nervous system involvement, from 4 months to 5 years of age. In the course of the study, a three-time single administration of RGX-121 to the cisterna magna or the lateral ventricle is planned in increasing doses, 1.3 × 10^10^ genome copies (GC)/g brain mass, 6.5 × 10^10^ GC/g brain mass, and 2.0 × 10^11^ GC/g brain mass, respectively [70]. The patients are assessed in this study for safety, tolerance and efficacy for 104 weeks after administration. Markers of the disease in serum, urine and cerebrospinal fluid are tested, and the development of the CNS (Bayley Scales of Infant and Toddler Development, BSID; Vineland Adaptive Behavior Scales, VABS) are assessed. Imaging diagnostics are also performed. The six patients who take RGX-121 in this study tolerate it well, and no severe drug-related adverse events were observed (as of September 2020) [73].

The second study (ClinicalTrials.gov Identifier: NCT04571970) targeted patients with severe Hunter syndrome aged 5 to 17. In this study, the vector administration route is also intracisternal or intracerebroventricular injection, but only once at a dose of 6.5 × 10^10^ GC/g brain mass. Similarly to the study mentioned earlier, the complete safety and efficacy evaluation will take 104 weeks from the agent’s application [71]. Both of these trials are still actively recruiting participants.

Adeno-associated viruses are currently the most optimal choice for delivering the correct gene to affected cells among the viral vectors available. Their ability to provide stable gene expression, deliver it to both dividing and non-dividing cells, and a low risk of mutagenesis make their use in clinical trials likely to increase. The apparent limitation of all viral vectors administered into the body is the risk of developing an immune response against them. However, the above-mentioned benefits of using AAVs outweigh their immunogenicity, which is not major anyway.

### 2.5. Non-Viral Vectors

One way to overcome the limitations of viral vectors in gene therapy is using other vectors that are not based on viral particles. Non-viral vectors include nanoemulsions, the transposon system with the Sleeping Beauty transposase or the electro gene transfer method [35,74]. They are not genotoxic but have limited efficiency in delivering the gene to cells and maintaining its expression.

In research on MPS II using non-viral vectors, preclinical trials have been carried out using only gene electrotransfer, i.e., a method of delivering exogenous molecules into cells through electrical pulses [75,76]. Friso et al. administered 50 µg of plasmid DNA containing cDNA for the *IDS* in 50 µL solution into quadriceps of mice. Hyaluronidase was injected into the muscle before administration of the plasmid itself to increase the effectiveness. The muscle was electrically stimulated with a current of 75 mA. The procedure proved to be very effective in protein production in the muscle, unfortunately without the proper activity of iduronate 2-sulphatase in the blood. Additionally, the authors observed a strong immune response against the recombinant protein [76]. Thus far, no other research group have performed studies with non-viral vectors in MPS II. It seems that their use may be limited due to the low level of expression and difficulties in delivering them to target tissues, especially the central nervous system. For this mucopolysaccharidosis, the use of viral vectors has so far been more optimal.

### 2.6. Genome Editing Methods

Another promising way to obtain the correct gene into cells is genome editing methods that have been intensively developed in the last decade [77]. Genome editing platforms applicable to therapeutic trials include zinc finger nucleases, transcription activator-like effector nucleases (TALENs), and the CRISPR/Cas9 system, and based on it: CRISPR/Cas9-based editors and CRISPR/Cas9-prime editing. Thus far, only ZFNs and CRISPR/Cas9 have been used in preclinical studies on mucopolysaccharidoses. The essence of these methods is to make breaks in double-strand DNA in a specific site in the genome and then repair the damage by two mechanisms: nonhomologous end joining (NHEJ) or homologous recombination (HR). The first mechanism is characterized by frequent insertions or deletions and is mainly used for reading frame correction. The second mechanism requires the template with the intended sequence and homologous sequences to the target site. Thanks to this, it is possible to correct even a single base without errors (single nucleotide variant, SNV) [48]. Zinc finger nucleases contain a domain that binds specifically to a particular DNA sequence and a nuclease domain derived from the restriction enzyme FokI [78]. In the CRISPR/Cas9 system, Cas9 nuclease binds to a target site through a short RNA sequence called guide RNA (gRNA). Additionally, in order to proper assembly, a short sequence called protospacer adjacent motif (PAM) is needed [79]. The most frequently used Cas9 nucleases in this technique come from the bacteria *Staphylococcus aureus* and *Streptococcus pyogenes* [80].

Laoharawee et al. conducted a preclinical study in a mouse MPS II model. They used zinc finger nucleases (ZFNs) to insert the human *IDS* gene in place of the first intron of the albumin gene *locus* in mouse hepatocytes. They applied AAV type 2/8 administered intravenously in increasing doses, 2.5 × 10^11^ vector genomes (vg), 5.0 × 10^11^ vg, and 1.5 × 10^12^ vg, respectively. The authors demonstrated normal activity of iduronate 2-sulphatase in blood and other tissues, as well as normalization of GAGs concentration in urine and organs. Additionally, they found that the applied therapy prevented the deterioration of neurocognitive functions in the examined mice [81].

Thanks to the positive results of research on model organisms, the first multicentre phase I/II clinical trial was launched in patients with MPS II (ClinicalTrials.gov Identifier: NCT03041324) using in vivo gene therapy based on genome editing [82]. This study uses SB-913 (Sangamo Therapeutics), which contains a zinc finger nuclease system, the correct *IDS* gene, and the AAV type 2/6 vector. The target site for *IDS* is the albumin *locus* in hepatocytes. The drug is administered intravenously in increasing doses. The study includes nine patients aged five years and older, divided into three cohorts depending on the dose. Currently, the recruitment for the trial is closed [83]. Sixteen weeks after the administration, in the middle dose group, a decrease in the concentration of GAGs in urine was observed, but no activity of the enzyme in the blood was found [84]. 

Genome editing methods are gaining significant interest due to their precision in reaching the target site in the genome. It seems that they will gain an advantage over other forms of gene therapy as they do not randomly integrate the correct gene but directly repair the defective sequence in its *locus* and provide long-stand gene expression. Genome editing systems, like their vectors, AAVs, are exogenous particles, and immune response may be developed against them. However, the benefits of using these precision genetic modification tools outweigh the potential limitations. A summary of the methods of gene therapy in mucopolysaccharidosis type II is presented in Table 2.

## 3. Challenges and Perspective

Gene therapy for mucopolysaccharidosis type II has made tremendous progress since the first studies on cell lines in the 1990s. However, there are still a few limitations that researchers dealing with this topic must face. First, many animal models differ from the in vivo conditions in the human body. The beneficial effects of the applied therapies in the mouse model, especially on the central nervous system, may not necessarily be reproduced in human studies due to the therapeutic threshold, which differs between species.

Another limitation is the risk of generating an immune response that mainly affects viruses but also elements of genome editing systems. AAVs seem to be the most optimal viral vector so far. However, neutralizing antibodies have been reported, and they lead the way to reduce the effectiveness of the entire therapy [85]. Additionally, cells that have already received the transgene can be eliminated by lymphocytes CD8+. Therefore, it is worth considering the use of immunosuppression in order to increase the effectiveness of subsequent gene therapy attempts [48,55]. A solution to this problem could be non-viral vectors, but so far, they have not been widely used in model organisms in MPS II, so further studies are needed to assess their efficacy and safety.

The use of retroviral and lentiviral vectors is likely to be abandoned in the near future due to their random integration into the genome and the risk of mutagenesis. AAVs seem to be the most optimal vectors. Combined with the precise genome editing method, they are currently the best form of gene therapy and the only one so far used in human clinical trials. It is possible that in the next few years, new clinical studies will be opened, and this process would be significantly accelerated by the positive results of phase I/II trials with RGX-121 and SB-913.

It is also worth mentioning additional therapy that may play a role in improving organ functions and quality of life in patients with Hunter syndrome soon. The process of autophagy, which is disturbed in lysosomal storage disorders, may become a potential therapeutic target. Maeda et al. [86] analysed the morphology of central nervous system cells in the MPS II mouse model. They showed an increased number of autophagy vesicles in an electron microscope and an increased concentration of the autophagy impairment markers—p62 protein and subunit c of mitochondrial ATP synthetase (SCMAS). The authors administered chloroquine orally at a dose of 10 mg/day for 25 weeks to improve the function of neurons. After this period, the vacuolization in neurons was significantly reduced compared to untreated individuals. Chloroquine can inhibit autophagy. Its use in MPS II patients may slow down neurodegenerative processes in the CNS. Researchers also tried to use verapamil and rapamycin (drugs that promote autophagy), but the study had to be discontinued due to serious adverse effects in mice [86].

## 4. Conclusions

Mucopolysaccharidosis type II, as a monogenic disease with relatively well-known pathophysiology, is an optimal example of the possibility of applying gene therapy. Over the past two decades, this technology has been advanced enough to open up clinical trials using the most promising methods—genome editing and adeno-associated viral vectors. Thus far, it has not been possible to come up with an ideal therapeutic agent. Additionally, gene therapy has some limitations: the immunogenicity of vectors and transgenic elements and the relatively high cost of developing this technology. However, comparing gene therapy to the current standard of treatment for Hunter syndrome—enzyme replacement therapy is essential. A single administration of a vector that will deliver the *IDS* gene to target cells and maintain a high expression level far exceeds the need for repeated intravenous administration of the enzyme. ERT applied intravenously is a heavy burden on the healthcare system and partially improves somatic symptoms without improving central nervous system function.

In conclusion, the further development of molecular engineering techniques and the ongoing clinical trials in humans will likely contribute to the broader use of gene therapy in mucopolysaccharidosis type II. Additionally, this will lead to faster achievement of therapeutic goals and improvement of patients’ quality of life.

## Figures and Tables

**Table 1:** Comparison of enzyme replacement therapy, gene therapy, and hematopoietic stem cell transplantation.

Features	Gene Therapy	Enzyme Replacement Therapy (ERT)	Hematopoietic Stem Cell Transplantation (HSCT)
The essence of the method	Delivery of the correct gene to cells by vector	Administration of the correct enzyme into the organism	Transplantation of donor cells with the correct gene
Number of therapeutic interventions	Single	Multiple	Single
The main advantages of the method	Stable gene expression, single application with a long-standing effect, improvement in the central nervous system (CNS) and other organs	Relatively safe route of administration (intravenous), the only Food and Drug Administration (FDA)-approved method so far	The ability to deliver the enzyme to the brain via monocytes, a single application
The main disadvantages of the method	The risk of developing an immune response against the vector and elements of the transgene, the risk of mutagenesis in the case of viral vectors	The need for multiple administration of the enzyme, the inability of the enzyme to cross the blood–brain barrier, the risk of developing neutralizing antibodies against the enzyme	Conditioning before HSCT, immunosuppression, the risk of graft vs. host disease (GvHD), limited gene expression in the central nervous system

**Table 2:** Comparison of current methods of gene therapy in mucopolysaccharidosis type II.

Features	Adeno-Associated Viruses	Lentiviruses	Retroviruses	Genome Editing	Non-Viral Vectors
The main advantages	Possibility of delivering the gene to non-dividing cells, present in the cell in the form of an episome, low immunogenicity, stable gene expression	Possibility of delivering the gene to non-dividing cells, stable gene expression	Stable gene expression	Ability to precise correction of gene abnormalities	No risk of mutagenesis, lower costs of the method
The main limitations	Immunogenicity, minor risk of mutagenesis	Random integration with the genome, risk of mutagenesis, immunogenicity	Random integration with the genome, risk of mutagenesis, immunogenicity	High costs of the method, immunogenicity	Difficulty in delivering the gene to the target cell, low gene expression, immunogenicity
Use in clinical trials in patients with mucopolysaccharidosis type II	Yes, currently, active trials [70,71]	No	Yes, trials closed [45]	Yes, currently, active trials [83]	No

## Data Availability

No new data were created or analyzed in this study. Data sharing is not applicable to this article.

## Abbreviations

AAV	Adeno-associated virus
AAVs	Adeno-associated viruses
BBB	Blood–brain barrier
CDNA	complementary DNA
CNS	Central nervous system
CRISPR	Clustered Regularly Interspaced Short Palindromic Repeat
CSF	Cerebrospinal fluid
DS	Dermatan sulphate
ERT	Enzyme replacement therapy
FDA	Food and Drug Administration
FGF	Fibroblast growth factor
GAGs	Glycosaminoglycans
GC	Genome copies
GvHD	Graft versus host disease
gRNA	guide RNA
HS	Heparan sulphate
HSCT	Hematopoietic stem cell transplantation
HR	Homologous recombination
iPSCs	induced pluripotent stem cells
KSPD	Kyoto Scale of Psychological Development 2001
LSDs	Lysosomal storage disorders
MPS I	Mucopolysaccharidosis type I
MPS II	Mucopolysaccharidosis type II
MPS IIIA	Mucopolysaccharidosis type IIIA
NGS	Next Generation Sequencing
NHEJ	Nonhomologous end joining
PAM	Protospacer adjacent motif
SCMAS	Subunit c of mitochondrial ATP synthetase
SNV	Single nucleotide variant
TALENs	Transcription activator-like effector nucleases
TLR4	Toll-like receptor 4
TNF-α	Tumour necrosis factor α
Vg	vector genomes
ZFNs	Zinc finger nucleases

## References

[1] Peters H., Ellaway C., Nicholls K., Reardon K., Szer J. (2020). Treatable lysosomal storage diseases in the advent of disease-specific therapy. Int. Med. J..

[2] Khan S.A., Peracha H., Ballhausen D., Wiesbauer A., Rohrbach M., Gautschi M., Mason R.W., Giugliani R., Suzuki Y., Orii K.E. (2017). Epidemiology of mucopolysaccharidoses. Mol. Genet. Metab..

[3] Manara R., Rampazzo A., Cananzi M., Salviati L., Mardari R., Drigo P., Tomanin R., Gasparotto N., Priante E., Scarpa M. (2010). Hunter syndrome in an 11-year old girl on enzyme replacement therapy with idursulfase: Brain magnetic resonance imaging features and evolution. J. Inherit. Metab. Dis..

[4] Tuschl K., Gal A., Paschke E., Kircher S., Bodamer O.A. (2005). Mucopolysaccharidosis type II in females: Case report and review of literature. Pediatr. Neurol..

[5] Sohn Y.B., Kim S.J., Park S.W., Park H.-D., Ki C.-S., Kim C.H., Huh S.W., Yeau S., Paik K.-H., Jin D.-K. (2010). A mother and daughter with the p.R443X mutation of mucopolysaccharidosis type II: Genotype and phenotype analysis. Am. J. Med. Genet. Part A.

[6] Kloska A., Jakóbkiewicz-Banecka J., Tylki-Szymańska A., Czartoryska B., Węgrzyn G. (2011). Female Hunter syndrome caused by a single mutation and familial XCI skewing: Implications for other X-linked disorders. Clin. Genet..

[7] Jurecka A., Krumina Z., Żuber Z., Różdżyńska-Świątkowska A., Kłoska A., Czartoryska B., Tylki-Szymańska A. (2012). Mucopolysaccharidosis type II in females and response to enzyme replacement therapy. Am. J. Med. Genet. Part A.

[8] Piña-Aguilar R.E., Zaragoza-Arévalo G.R., Rau I., Gal A., Alcántara-Ortigoza M.A., López-Martínez M.S., Santillán-Hernández Y. (2013). Mucopolysaccharidosis type II in a female carrying a heterozygous stop mutation of the iduronate-2-sulfatase gene and showing a skewed X chromosome inactivation. Eur. J. Med. Genet..

[9] Lonardo F., Di Natale P., Lualdi S., Acquaviva F., Cuoco C., Scarano F., Maioli M., Pavone L.M., Di Gregorio G., Filocamo M. (2014). Mucopolysaccharidosis type II in a female patient with a reciprocal X;9 translocation and skewed X chromosome inactivation. Am. J. Med. Genet. Part A.

[10] Hunter C. (1917). A Rare Disease in Two Brothers. Proc. R. Soc. Med..

[11] D’Avanzo F., Rigon L., Zanetti A., Tomanin R. (2020). Mucopolysaccharidosis Type II: One Hundred Years of Research, Diagnosis, and Treatment. Int. J. Mol. Sci..

[12] McBride K.L., Berry S.A., Braverman N. (2020). ACMG Therapeutics Committee. Treatment of mucopolysaccharidosis type II (Hunter syndrome): A Delphi derived practice resource of the American College of Medical Genetics and Genomics (ACMG). Genet. Med..

[13] Stapleton M., Kubaski F., Mason R.W., Yabe H., Suzuki Y., Orii K.E., Orii T., Tomatsu S. (2017). Presentation and treatments for Mucopolysaccharidosis Type II (MPS II.; Hunter Syndrome). Expert Opin. Orphan Drugs.

[14] Bellesso S., Salvalaio M., Lualdi S., Tognon E., Costa R., Braghetta P., Giraudo C., Stramare R., Rigon L., Filocamo M. (2018). FGF signaling deregulation is associated with early developmental skeletal defects in animal models for mucopolysaccharidosis type II (MPSII). Hum. Mol. Genet..

[15] Fecarotta S., Gasperini S., Parenti G. (2018). New treatments for the mucopolysaccharidoses: From pathophysiology to therapy. Ital. J. Pediatr..

[16] Braun S.E., Pan D., Aronovich E.L., Jonsson J.J., McIvor R.S., Whitley C.B. (1996). Preclinical studies of lymphocyte gene therapy for mild Hunter syndrome (mucopolysaccharidosis type II). Hum. Gene Ther..

[17] Seo J.H., Okuyama T., Shapiro E., Fukuhara Y., Kosuga M. (2020). Natural history of cognitive development in neuronopathic mucopolysaccharidosis type II (Hunter syndrome): Contribution of genotype to cognitive developmental course. Mol. Genet. Metab. Rep..

[18] Zanetti A., D’Avanzo F., Bertoldi L., Zampieri G., Feltrin E., De Pascale F., Rampazzo A., Forzan M., Valle G., Tomanin R. (2020). Setup and validation of a targeted NGS approach for the diagnosis of lysosomal storage disorders. J. Mol. Diagn..

[19] Brusius-Facchin A.C., Siebert M., Leão D., Malaga D.R., Pasqualim G., Trapp F., Matte U., Giugliani R., Leistner-Segal S. (2019). Phenotype-oriented NGS panels for mucopolysaccharidoses: Validation and potential use in the diagnostic flowchart. Genet. Mol. Biol..

[20] Fratantoni J.C., Hall C.W., Neufeld E.F. (1968). Hurler and Hunter syndromes: Mutual correction of the defect in cultured fibroblasts. Science.

[21] Hasilik A., Neufeld E.F. (1980). Biosynthesis of lysosomal enzymes in fibroblasts. Phosphorylation of mannose residues. J. Biol. Chem..

[22] Higuchi T., Shimizu H., Fukuda T., Kawagoe S., Matsumoto J., Shimada Y., Kobayashi H., Ida H., Ohashi T., Morimoto H. (2012). Enzyme replacement therapy (ERT) procedure for mucopolysaccharidosis type II (MPS II) by intraventricular administration (IVA) in murine MPS II. Mol. Genet. Metab..

[23] Sohn Y.B., Ko A.R., Seong M.R., Lee S., Kim M.R., Cho S.Y., Kim J.S., Sakaguchi M., Nakazawa T., Kosuga M. (2018). The efficacy of intracerebroventricular idursulfase-beta enzyme replacement therapy in mucopolysaccharidosis II murine model: Heparan sulfate in cerebrospinal fluid as a clinical biomarker of neuropathology. J. Inherit. Metab. Dis..

[24] Nan H., Park C., Maeng S. (2020). Mucopolysaccharidoses I and II: Brief Review of Therapeutic Options and Supportive/Palliative Therapies. BioMed Res. Int..

[25] Muenzer J., Hendriksz C.J., Fan Z., Vijayaraghavan S., Perry V., Santra S., Solanki G.A., Mascelli M.A., Pan L., Wang N. (2016). A phase I/II study of intrathecal idursulfase-IT in children with severe mucopolysaccharidosis II. Genet. Med..

[26] Seo J.H., Kosuga M., Hamazaki T., Shintaku H., Okuyama T. (2021). Impact of intracerebroventricular enzyme replacement therapy in patients with neuronopathic mucopolysaccharidosis type II. Mol. Ther. Methods Clin. Dev..

[27] Sato Y., Okuyama T. (2020). Novel Enzyme Replacement Therapies for Neuropathic Mucopolysaccharidoses. Int. J. Mol. Sci..

[28] Kida S., Kinoshita M., Tanaka S., Okumura M., Koshimura Y., Morimoto H. (2019). Non-clinical evaluation of a blood-brain barrier-penetrating enzyme for the treatment of mucopolysaccharidosis type I. Mol. Genet. Metab..

[29] Okuyama T., Eto Y., Sakai N., Nakamura K., Yamamoto T., Yamaoka M., Ikeda T., So S., Tanizawa K., Sonoda H. (2021). A Phase 2/3 Trial of Pabinafusp Alfa, IDS Fused with Anti-Human Transferrin Receptor Antibody, Targeting Neurodegeneration in MPS-II. Mol. Ther..

[30] JCR Pharma: Crossing Oceans and the Blood-Brain Barrier. https://invivo.pharmaintelligence.informa.com/IV124753/JCR-Pharma-Crossing-Oceans-And-The-Blood-Brain-Barrier.

[31] ClinicalTrials.gov A Study of DNL310 in Pediatric Subjects with Hunter Syndrome. https://clinicaltrials.gov/ct2/show/NCT04251026?term=NCT04251026&draw=2&rank=1.

[32] Tong W., Dwyer C.A., Thacker B.E., Glass C.A., Brown J.R., Hamill K., Moremen K.W., Sarrazin S., Gordts P., Dozier L.E. (2017). Guanidinylated Neomycin Conjugation Enhances Intranasal Enzyme Replacement in the Brain. Mol. Ther..

[33] Lampe C., Bosserho A.-K., Burton B.K., Giugliani R., de Souza C.F., Bittar C., Muschol N., Olson R., Mendelsohn N.J. (2014). Long-term experience with enzyme replacement therapy (ERT) in MPS II patients with a severe phenotype: An international case series. J. Inherit. Metab. Dis..

[34] Araya K., Sakai N., Mohri I., Kagitani-Shimono K., Okinaga T., Hashii Y., Ohta H., Nakamichi I., Aozasa K., Taniike M. (2009). Localized donor cells in brain of a Hunter disease patient after cord blood stem cell transplantation. Mol. Genet. Metab..

[35] Kaufmann K.B., Büning H., Galy A., Schambach A., Grez M. (2013). Gene therapy on the move. EMBO Mol. Med..

[36] Schuh R.S., de Carvalho T.G., Giugliani R., Matte U., Baldo G., Teixeira H.F. (2018). Gene editing of MPS I human fibroblasts by co-delivery of a CRISPR/Cas9 plasmid and a donor oligonucleotide using nanoemulsions as nonviral carriers. Eur. J. Pharm. Biopharm..

[37] Blaese R.M., Culver K.W., Miller A.D., Carter C.S., Fleisher T., Clerici M., Shearer G., Chang L., Chiang Y., Tolstoshev P. (1995). T lymphocyte-directed gene therapy for ADA- SCID: Initial trial results after 4 years. Science.

[38] Wilson J.M. (2005). Gendicine: The first commercial gene therapy product. Hum. Gene Ther..

[39] Zhang W.W., Li L., Li D., Liu J., Li X., Li W., Xu X., Zhang M.J., Chandler L.A., Lin H. (2018). The First Approved Gene Therapy Product for Cancer Ad-p53 (Gendicine): 12 Years in the Clinic. Hum. Gene Ther..

[40] Miller N. (2012). Glybera and the future of gene therapy in the European Union. Nat. Rev. Drug Discov..

[41] Ylä-Herttuala S. (2012). Endgame: Glybera finally recommended for approval as the first gene therapy drug in the European union. Mol. Ther..

[42] Mendell J.R., Al-Zaidy S.A., Rodino-Klapac L.R., Goodspeed K., Gray S.J., Kay C.N., Boye S.L., Boye S.E., George L.A., Salabarria S. (2021). Current Clinical Applications of In Vivo Gene Therapy with AAVs. Mol. Ther..

[43] Hong Y., Yu S.S., Kim J.M., Lee K., Na Y.S., Whitley C.B., Sugimoto Y., Kim S. (2003). Construction of a high efficiency retroviral vector for gene therapy of Hunter’s syndrome. J. Gene Med..

[44] Sawamoto K., Stapleton M., Alméciga-Díaz C.J., Espejo-Mojica A.J., Losada J.C., Suarez D.A., Tomatsu S. (2019). Therapeutic Options for Mucopolysaccharidoses: Current and Emerging Treatments. Drugs.

[45] ClinicalTrials.gov Phase I/II Study of Retroviral-Mediated Transfer of Iduronate-2-Sulfatase Gene into Lymphocytes of Patients with Mucopolysaccharidosis II (Mild Hunter Syndrome). https://clinicaltrials.gov/ct2/show/NCT00004454.

[46] Wada M., Shimada Y., Iizuka S., Ishii N., Hiraki H., Tachibana T., Maeda K., Saito M., Arakawa S., Ishimoto T. (2020). Gene Therapy Treats Bone Complications of Mucopolysaccharidosis Type II Mouse Models through Bone Remodeling Reactivation. Mol. Ther. Methods Clin. Dev..

[47] Jung S.C., Park E.S., Choi E.N., Kim C.H., Kim S.J., Jin D.K. (2010). Characterization of a novel mucopolysaccharidosis type II mouse model and recombinant AAV2/8 vector-mediated gene therapy. Mol. Cells.

[48] Poletto E., Baldo G., Gomez-Ospina N. (2020). Genome Editing for Mucopolysaccharidoses. Int. J. Mol. Sci..

[49] Yamanaka S. (2012). Induced pluripotent stem cells: Past, present, and future. Cell Stem Cell.

[50] Sakuma T., Barry M.A., Ikeda Y. (2012). Lentiviral vectors: Basic to translational. Biochem. J..

[51] Milone M.C., O’Doherty U. (2018). Clinical use of lentiviral vectors. Leukemia.

[52] Wakabayashi T., Shimada Y., Akiyama K., Higuchi T., Fukuda T., Kobayashi H., Eto Y., Ida H., Ohashi T. (2015). Hematopoietic Stem Cell Gene Therapy Corrects Neuropathic Phenotype in Murine Model of Mucopolysaccharidosis Type II. Hum. Gene Ther..

[53] ClinicalTrials.gov Gene Therapy with Modified Autologous Hematopoietic Stem Cells for the Treatment of Patients with Mucopolysaccharidosis Type I, Hurler Variant (TigetT10_MPSIH). https://clinicaltrials.gov/ct2/show/NCT03488394.

[54] ClinicalTrials.gov Gene Therapy with Modified Autologous Hematopoietic Stem Cells for Patients with Mucopolysaccharidosis Type IIIA. https://clinicaltrials.gov/ct2/show/NCT04201405.

[55] Colella P., Ronzitti G., Mingozzi F. (2018). Emerging Issues in AAV-Mediated In Vivo Gene Therapy. Mol. Ther. Methods Clin. Dev..

[56] Balakrishnan B., Jayandharan G.R. (2014). Basic biology of adeno-associated virus (AAV) vectors used in gene therapy. Curr. Gene Ther..

[57] Calcedo R., Morizono H., Wang L., McCarter R., He J., Jones D., Batshaw M.L., Wilson J.M. (2011). Adeno-associated virus antibody profiles in newborns, children, and adolescents. Clin. Vaccine Immunol..

[58] Smith R.H. (2008). Adeno-associated virus integration: Virus versus vector. Gene Ther..

[59] McCarty D.M., Young S.M., Samulski R.J. (2004). Integration of Adeno-Associated Virus (AAV) and Recombinant AAV Vectors. Annu. Rev. Genet..

[60] Donsante A., Vogler C., Muzyczka N., Crawford J.M., Barker J., Flotte T., Campbell-Thompson M., Daly T., Sands M.S. (2001). Observed incidence of tumorigenesis in long-term rodent studies of rAAV vectors. Gene Ther..

[61] Rosas L.E., Grieves J.L., Zaraspe K., La Perle K.M.D., Fu H., McCarty D.M. (2012). Patterns of scAAV vector insertion associated with oncogenic events in a mouse model for genotoxicity. Mol. Ther..

[62] Daly T.M., Okuyama T., Vogler C., Haskins M.E., Muzyczka N., Sands M.S. (1999). Neonatal intramuscular injection with recombinant adeno-associated virus results in prolonged beta-glucuronidase expression in situ and correction of liver pathology in mucopolysaccharidosis type VII mice. Hum. Gene Ther..

[63] Davidson B.L., Stein C.S., Heth J.A., Martins I., Kotin R.M., Derksen T.A., Zabner J., Ghodsi A., Chiorini J.A. (2000). Recombinant adeno-associated virus type 2, 4, and 5 vectors: Transduction of variant cell types and regions in the mammalian central nervous system. Proc. Natl. Acad. Sci. USA.

[64] Stein C.S., Ghodsi A., Derksen T., Davidson B.L. (1999). Systemic and central nervous system correction of lysosomal storage in mucopolysaccharidosis type VII mice. J. Virol..

[65] Sands M.S., Davidson B.L. (2006). Gene therapy for lysosomal storage diseases. Mol. Ther..

[66] Cardone M., Polito V.A., Pepe S., Mann L., D’Azzo A., Auricchio A., Ballabio A., Cosma M.P. (2006). Correction of Hunter syndrome in the MPSII mouse model by AAV2/8-mediated gene delivery. Hum. Mol. Genet..

[67] Hinderer C., Katz N., Louboutin J.P., Bell P., Yu H., Nayal M., Kozarsky K., O’Brien W.T., Goode T., Wilson J.M. (2016). Delivery of an Adeno-associated virus vector into cerebrospinal fluid attenuates central nervous system disease in mucopolysaccharidosis type II mice. Hum. Gene Ther..

[68] Laoharawee K., Podetz-Pedersen K.M., Nguyen T.T., Evenstar L.B., Kitto K.F., Nan Z., Fairbanks C.A., Low W.C., Kozarsky K.F., McIvor R.S. (2017). Prevention of neurocognitive deficiency in mucopolysaccharidosis type II mice by central nervous system-directed, AAV9-mediated iduronate sulfatase gene transfer. Hum. Gene Ther..

[69] Motas S., Haurigot V., Garcia M., Marcó S., Ribera A., Roca C., Sánchez X., Sánchez V., Molas M., Bertolin J. (2016). CNS-directed gene therapy for the treatment of neurologic and somatic mucopolysaccharidosis type II (Hunter syndrome). JCI Insight.

[70] ClinicalTrials.gov RGX-121 Gene Therapy in Patients with MPS II (Hunter Syndrome). https://clinicaltrials.gov/ct2/show/NCT03566043.

[71] ClinicalTrials.gov RGX-121 Gene Therapy in Children 5 Years of Age and Over with MPS II (Hunter Syndrome). https://clinicaltrials.gov/ct2/show/NCT04571970.

[72] Cowley K., Falabella P., Pakola S., Nevoret M.-L. (2021). RGX-121 gene therapy for severe mucopolysaccharidosis type II (MPS II): A clinical program to address central nervous system manifestations. Mol. Genet. Metab..

[73] Nevoret M.-L., Escolar M., Ficicioglu C., Giugliani R., Harmatz P., Cho Y., Phillips D., Falabella P. (2021). RGX-121 gene therapy for severe mucopolysaccharidosis type II (MPS II): Interim results of an ongoing first in human trial. Mol. Genet. Metab..

[74] Tomanin R., Friso A., Alba S., Piller Puicher E., Mennuni C., La Monica N., Hortelano G., Zacchello F., Scarpa M. (2002). Non-viral transfer approaches for the gene therapy of mucopolysaccharidosis type II (Hunter syndrome). Acta Paediatrica.

[75] Mir L.M., Moller P.H., André F., Gehl J. (2005). Electric pulse-mediated gene delivery to various animal tissues. Adv. Genet..

[76] Friso A., Tomanin R., Zanetti A., Mennuni C., Calvaruso F., La Monica N., Marin O., Zacchello F., Scarpa M. (2008). Gene therapy of Hunter syndrome: Evaluation of the efficiency of muscle electro gene transfer for the production and release of recombinant iduronate-2-sulfatase (IDS). Biochim. Biophys. Acta Mol. Basis Dis..

[77] Bak R.O., Gomez-Ospina N., Porteus M.H. (2018). Gene Editing on Center Stage. Trends Genet..

[78] Urnov F.D., Rebar E.J., Holmes M.C., Zhang H.S., Gregory P.D. (2010). Genome editing with engineered zinc finger nucleases. Nat. Rev. Genet..

[79] Doudna J.A., Charpentier E. (2014). The new frontier of genome engineering with CRISPR-Cas9. Science.

[80] Ran F.A., Cong L., Yan W.X., Scott D.A., Gootenberg J.S., Kriz A.J., Zetsche B., Shalem O., Wu X., Makarova K.S. (2015). In vivo genome editing using Staphylococcus aureus Cas9. Nature.

[81] Laoharawee K., DeKelver R.C., Podetz-Pedersen K.M., Rohde M., Sproul S., Nguyen H.O., Nguyen T., St Martin S.J., Ou L., Tom S. (2018). Dose-Dependent Prevention of Metabolic and Neurologic Disease in Murine MPS II by ZFN-Mediated In vivo Genome Editing. Mol. Ther..

[82] Nature America, Inc. (2018). First in vivo human genome editing trial. Nat. Biotechnol..

[83] ClinicalTrials.gov Ascending Dose Study of Genome Editing by the Zinc Finger Nuclease (ZFN) Therapeutic SB-913 in Subjects with MPS II. https://clinicaltrials.gov/ct2/show/NCT03041324.

[84] Sheridan C. (2018). Sangamo’s landmark genome editing trial gets mixed reception. Nat. Biotechnol..

[85] Boutin S., Monteilhet V., Veron P., Leborgne C., Benveniste O., Montus M.F., Masurier C. (2010). Prevalence of serum IgG and neutralizing factors against adeno-associated virus (AAV) types 1, 2, 5, 6, 8, and 9 in the healthy population: Implications for gene therapy using AAV vectors. Hum. Gene Ther..

[86] Maeda M., Seto T., Kadono C., Morimoto H., Kida S., Suga M., Nakamura M., Kataoka Y., Hamazaki T., Shintaku H. (2019). Autophagy in the Central Nervous System and Effects of Chloroquine in Mucopolysaccharidosis Type II Mice. Int. J. Mol. Sci..

